# Obstetrical Management of an Extremely Overweight Pregnant Woman (184 kg bw) with Special Attention on Thromboprophylaxis

**DOI:** 10.1155/2013/689549

**Published:** 2013-03-04

**Authors:** Boldizsar Horváth, Judit Skrapits, József Bódis

**Affiliations:** ^1^Faculty of Health Sciences, University of Pécs, Vörösmarty ut 3., Pécs 7621, Hungary; ^2^Markusovszky Teaching Hospital, Markusovszky u. 3., Szombathely 9700, Hungary

## Abstract

The 27-year-old pregnant woman has been overweight since her childhood. Endocrinological assessments did not confirm hormonal disease. Her pregnancy was without complication. A signs of intrauterine distress were observed and elective caesarean section was performed under heparin protection because of anatomy unsuitable for delivery per vias naturals. The mother's bodyweight was 184 kg. By monitoring the change in fX activity LMWH treatment (Enoxaparin) initiated with a dose of 120 mg twice daily and then the dose was gradually elevated to 200 mg twice daily thereby achieving the lower range of the desired therapeutic effect. Apart from mild disorder of wound healing, the recovery was free of complication. The patient suffered from thrombophilia (extremely overweight, pregnant, thrombophlebitis under the knee, surgery, and postoperative immobilization). In case of quite extreme bodyweight there is no dosage recommendation or clinical practice for LMWH. Because of the extreme overweight and the therapeutic dose titration test of heparin, monitoring of fX activity by measurement of inhibition, dosage of heparin other than the recommended (abdominal wall instead of upper arm SC), and the very fluctuating heparin dosage which is well correlating with clinical practice, it is reasonably expected that this case will take interest.

## 1. Introduction

Overweight and obesity have proven to be the most significant health problem of the present time and the future. Data from the WHO have shown that overweight and obesity are the second most important preventable risk factor after smoking [1]. Obesity is known to be harmful to health as obese people more often suffer from diseases that increase premature mortality which is on one hand the consequence of direct effects and on the other hand can be explained by the other diseases that develop [2–4]. As obesity may be associated with many maternal and foetal/neonatal complications, it is advisable that the gynaecologist informs his/her patient of the relevant risks preferably before the obese woman gets pregnant. The higher the patient's BMI (Body Mass Index) is, the greater the probability of complication is [5–14]. In obese pregnant women (BMI >30 kg/m^2^) the incidence of gestational diabetes can be even 20 times the value measured in mothers with physiological bodyweight [3, 7, 13, 14]. The incidence of hypertension and preeclampsia is 2.2–21.4 and 9.7 times higher, respectively, compared to women with normal bodyweight. Obesity also significantly increases the risk of thromboembolic diseases [7, 15–19]. The risk of perinatal mortality is 2.5 to 3.4 times higher in overweight and obese pregnant women, respectively, compared to the mothers with physiological bodyweight [2, 4, 5, 8, 18–20].

Our case was extremely obese. The value of the so-called grade IV obesity is above 40 based on BMI, and our patient's bodyweight was 184 kg equivalent to 59.4! BMI. This overweight may raise medicinal, management, and technical-logistical problems. In our case—due to the lack of experience with the management of such patients—selection of the efficient thromboprophylaxis seemed critical. We could examine and follow the efficiency of heparin therapy adjusted to bodyweight in an extreme situation (as far as we know there is a very few healthcare providers that can deal with such situation); this is why we believe it is important to report our experiences.

## 2. Case Study

G.O. a 27-year-old pregnant woman has been overweight since her childhood. Multiple endocrinological assessments did not confirm hormonal disease. She has suffered from moderate bronchial asthma since her childhood but she did not have any other diseases. She did not have metabolic syndrome since the laboratory tests performed one year before she became pregnant did not indicate this (lack of insulin resistance, se. Cholesterol 5.3 mmol/L (ref. 3.9–5.2), se. HDL 2.0 mmol/L (0.9−), se LDL 2.5 mmol/L (0.1–3.4), se. Triglyceride 1.7 mmol/L (0.5–2.3)). Her cycles were regular; she became pregnant in the 4th cycle without contraception. At this time her bodyweight was approximately 174 kg; uxor was 62 kg. Her data showed that the pregnancy progressed normally; oral glucose tolerance test performed at week 27 confirmed normal carbohydrate metabolism (after administration of 75 g carbohydrate her blood glucose level was 5.0–7.8–6.4 mmol/L).

**Figure 1:**
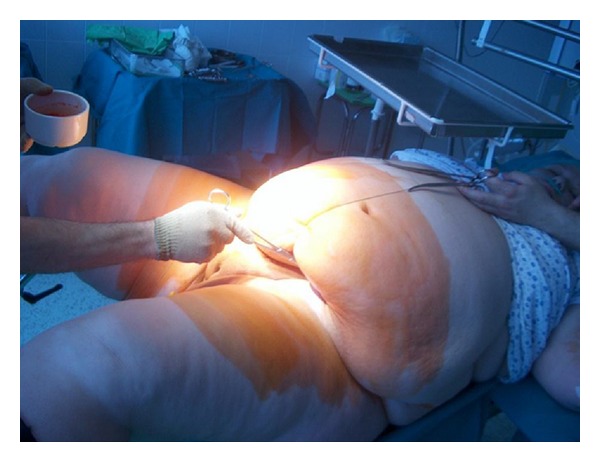
Preoperative preparation release of hanging abdomen to open the abdomen.

**Figure 2:**
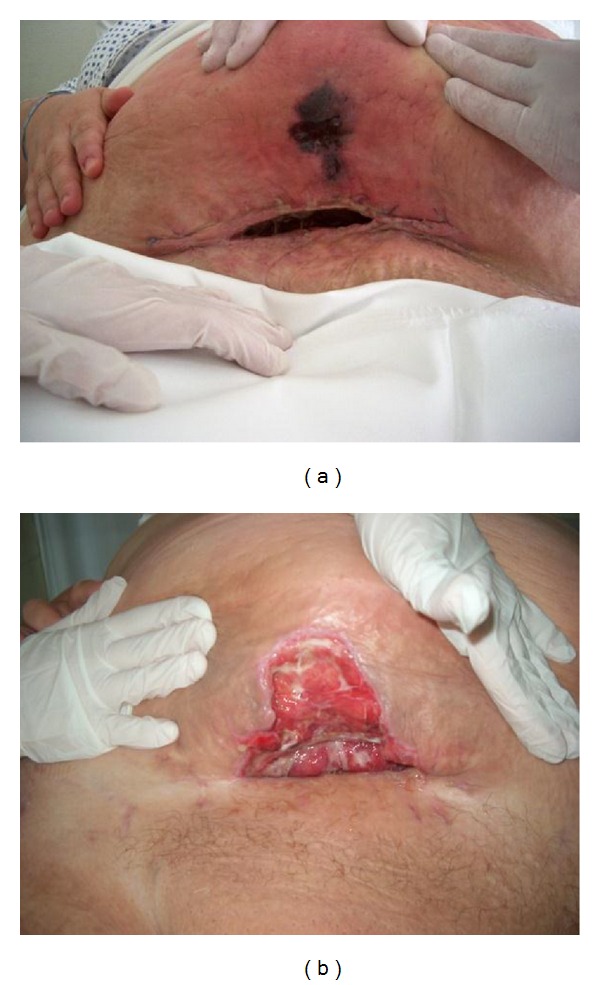
Wound healing on days 10 and 21 after surgery.

The pregnant woman treated in another institute was referred to our ward by her public health nurse due to hypertension near the end of pregnancy. Her blood pressure was 150/100 mm Hg at admission but no proteinuria was found. Untreated thrombophlebitis developing within few days below her right knee was found; additionally we recorded normal pregnancy. In addition to antihypertensive treatment and administration of diosmin (Detralex) and *α*-amino-benzyl-penicillin (Ampicillin), we applied heparin therapy. Based on thromboembolic risk assessment we categorized our patient into “very high risk group” when 1 to 1.5 mg/kg bw low molecular weight heparin (LMWH) twice daily is recommended. At this time our patient's bodyweight was 184 kg. We did not have experience with doses to be administered in case of such high bodyweight and we did not find relevant data even in the literature. We prescribed 120 mg Enoxaparin Sodium (Clexane) bid and monitoring of heparin therapy (Siemens Berichrom Heparin Calculator). The target was to achieve at least 0.5 anti-Xa activity four hours after injection [21–24]. After gradual increase of Clexane dose (2 × 160, 2 × 180, and 2 × 200 mg/day!), we measured therapeutic level on the fourth day (Table 1). Due to the extremely robust abdominal wall, the patient was administered heparin into the subcutaneous region of her shoulder for better absorption. After few days observation elective caesarean section was applied because of symptoms of mild intrauterine distress and the anatomy unsuitable for delivery through natural way (cervix cannot be found either digitally or with exploration). Surgery was performed under spinal anaesthesia according to Misgav-Ladach method in agreement with the patient because we found that her abdominal wall is the less thick in the abdominal fold (approx. 8 cm, in whole it was 16 cm at the level of linea alba inferior) (Figure 1). At the beginning of surgery the assistant standing at the head of the patient kept the pendulum abdomen above the planned surgical site and maintained its position by equipment during surgery by placement of three stitches—forming a bow (Figure 1). During the uneventful surgery a healthy female neonate was born with 2950 g bodyweight and with APGAR value of 8–10. Administration of 200 mg LMWH bid was discontinued prior to surgery later than recommended by the guidelines (10 h) and continued earlier 6 hours after surgery and mechanic thromboprophylaxis was also applied. Our patient who was very cooperative was mobilized 20 hours after surgery with no haemorrhagic complications observed. Despite perioperative antibiotic prophylaxis, our patient got fever on day 6 after surgery and infection of the abdominal wound was observed in the adipose layer. Wound toilet was applied twice daily (Figure 2). On day 12 of patient care she was discharged and instructed to return for wound treatment every day and self-administer 2 × 200 mg heparin bid, and weekly measurement of heparin level was required. Anti-fX activity confirmed therapeutic level while on day 32 after surgery the value was only 0.2 suddenly. It turned out that patient self-administered heparin later (she fell asleep) and the usual heparin measurement occurred 2 hours after administration of the drug (Table 1). Wound treatment was applied until week 4 after surgery and heparin treatment was given for 2 months. Week 6 follow-up examination confirmed normal status aside from the extreme bodyweight of the patient (173 kg bw).

## 3. Discussion

The thromboembolic disease is a multicausal condition; few components of it could be observed in our case [2, 15, 16]. Obese patients significantly increase the risk of venous thromboembolic event; moreover, extremely obese people are categorized as “very high risk” patients [25–27]. Relative risk of thromboembolic complications during pregnancy is 1/1000–2000 deliveries, that is, five to fifteen times increase in risk, and the risk is increased with additional 2-3 times after delivery. Caesarean section alone represents approximately 5 times higher risk compared to vaginal delivery. In each case when the patient is in bed rest (during and after surgery) compression stockings and/or prophylactic anticoagulation (primarily low molecular weight heparin) is recommended both for prophylaxis and treatment of thromboembolic diseases (recommendation level “2C”) [17, 25–28].

During caesarean section the literature prefers regional anaesthesia to intratracheal narcosis because of the more frequent complications with the latter. Concerning the type of abdominal section—due to higher incidence of wound healing disorders—individual consideration should be made based on discussion with the mother and at least a single-shot antibiotic prophylaxis is necessary after cutting the umbilicus, which we applied [29]. Efficient thrombosis prophylaxis is recommended during the postoperative period and due to the higher incidence of subinvolution of uterus administration of uterotonic agent is recommended in the puerperal period [9]. 

Our patient suffered from combined (acquired) thrombophilia (extremely obese, pregnant, thrombophlebitis below the knee, then surgery, and postoperative immobilization). In case of such extreme bodyweight noany clinical experience or dosage recommendation for LMWH was available [17, 21, 22, 25, 26]. Heparin therapy has demonstrated to be efficient despite the route of administration other than the recommendations (upper arm SC instead of abdomen SC) and extreme obesity. This was confirmed by measurement of change in fX activation. In this rare bodyweight category treatment with Enoxaparin 2 × 1 to 1.5 mg/kg bw bid is the correct dosage.

## Figures and Tables

**Table 1:** Pre-, peri-, and postoperative days the examine and follow the efficiency of heparin therapy by monitoring of anti-Xa activity.

Days	Sd. 38 Day 6	Opus Day 1*	Opus Day 0**	Postop. Hour 6	Postop. Day 2	Puerp. Day 6	Puerp. Day 32***	Puerp. Day 40
MWH (Clexane) mg	2 × 160	2 × 180	2 × 200	2 × 200	2 × 200	2 × 200	2 × 220	2 × 200
Heparin level IU/mL aXf.****	0.01	0.45	0.45	0.45	0.55	0.55	0.20	0.60
Comment	Hospital admission	Perioperative period			Self-administer bid	Infection	Faulty heparin measurement	Recovery

*Due to the extremely robust abdominal wall the patient was administered heparin into the subcutaneous region of her shoulder for better absorption.

**At time of performing caesarean section.

***The patient self-administered heparin later (she fell asleep) and measurement occurred 2 h after administer LMWH.

****The target was to achieve at least 0.5 anti-Xa activity four hours after injection.
